# Can the organization of health resource integration be analyzed in terms of the current state of unmet demand for health services? take the health needs of the elderly in a place in zhejiang province, china, as an example

**DOI:** 10.1186/s12875-022-01893-7

**Published:** 2022-11-19

**Authors:** Li Gao, Bing Wang, Xiaohong Yang, Yongliang Pan, Wenming Feng, Xuedan Pei, Yanfang Fan, Bing Liu

**Affiliations:** 1 Department of Medicine, Huzhou University, Huzhou, 313000 China; 2 Huzhou First People’s Hospital, Huzhou, China

**Keywords:** Health service demand side, Supply and demand balance, General practitioner, Elderly, Integrated Health Service System

## Abstract

**Background:**

The aging of the population has made the health problems of the elderly increasingly prominent, and their health needs are increasing. Existing studies on health resource integration approaches are mostly incomplete in assessing the health service capacity from the perspective of the health service provider.

**Objective:**

The unmet health needs of the elderly were sampled and analyzed from the perspective of health service demanders. To explore how to build an integrated medical organization structure to better meet the health needs of the elderly.

**Methods:**

A whole-group sampling method was used to conduct a questionnaire survey of 1527 older adults in N district of H city, Zhejiang province, China, to cross-sectionally analyze their current status of unmet health needs.

**Results:**

The survey and analysis found that the needs of the elderly in this community to obtain disease-related knowledge, rational exercise, a healthy diet, and access to health information were not met. There were more patients with chronic diseases, and the top three chronic disease prevalence rates were hypertension (40.2%), dyslipidemia (8.4), and diabetes (7%). Chronic disease co-morbidities accounted for 13.3%.

**Conclusion:**

The relatively independently set up health service system at the present stage in China can no longer fully meet the health needs of the elderly, and the health service providers should provide integrated and continuous health services to meet the needs of whole-cycle health management. Therefore, we believe that effectively integrating various health service providers in the region and building an integrated health service organization with general practitioners as the core may be a solution to the current situation of unmet health needs of the elderly.

**Supplementary Information:**

The online version contains supplementary material available at 10.1186/s12875-022-01893-7.

## Introduction

According to the results of China's seventh population census in 2020, the population aged 60 and above was 260 million, accounting for 18.7% of the total population, far exceeding the 10% aging standard stipulated by the United Nations. China has entered a seriously aging society. As the elderly population grows, so do the health needs of the elderly. To assess whether the capacity of primary health care services can meet the health needs of the elderly, the Chinese health administration has proposed to evaluate resource allocation-type indicators such as the number of beds per 1000 population and the health care ratio [1]. But such evaluation indicators are conducted only from the perspective of the supply side of health services, resulting in the demands of the demand side of health services not being fully identified and met. With the emergence of disadvantages such as fragmentation of services and low utilization of resources in the tertiary health care service system, the Chinese government has proposed relying on the community to provide comprehensive health management services for the elderly, drawing on the forms of health care in countries such as the United States [2] and the United Kingdom [3]. However, community-based health support services for the elderly are still in the exploratory stage. The number of health support services provided by the community accounts for less than 50% of all services. The content is mainly health education and knowledge propaganda in a single form, which cannot fully meet the health service needs of the elderly [4]. The "*Health China 2030*" plan outlines the need to promote the construction of a medical and health service system for the elderly and strengthen the health management of the elderly. At the same time, it is emphasized that building a high-quality and efficient integrated medical and health service system is a crucial initiative to "build a healthy China and achieve health for all" [5]. Integrative care has been recognized as improving access, quality, and continuity of services more effectively [6]. According to the World Health Organization's definition, integrated health care is a continuum of health promotion, disease prevention, diagnosis, treatment, and rehabilitation services provided by different levels of the health system through collaboration and according to the needs of people at different life stages [7]. In this context, Zhejiang Province, China, as one of the first practitioners of integrated medical service system construction, has accumulated specific experience building an integrated medical service system and created the "Zhejiang model" with local characteristics [8]. H City also actively responded to the national policy and innovatively proposed the joint model of the urban medical community [9]. Domestic and foreign research on organizing and structuring an integrated health care service system is mainly carried out from various dimensions such as institutional integration, organizational integration, professional integration, service integration, core function integration, etc. Among them, regarding how to carry out organizational integration, existing research mainly integrates various types of medical and health institutions at all levels, community institutions, voluntary organizations, and other organizations according to their functional positioning from the perspective of health service providers [10]. Evaluation metrics such as the number of two-week outpatient visits, two-week hospitalizations, etc., are incomplete in assessing whether the health needs of a population are being adequately met through simple per capitalization or capita living space. The existing organizational structure is also poorly coordinated, and older adults have many unmet needs. Therefore, to better build an integrated medical and health service system and adapt the existing integrated medical organization structure to the needs of the elderly, we ask if it is possible to analyze and evaluate the form of health resource integration in terms of the current state of unmet demand for health services? This study conducted a questionnaire survey on older adults in N district of H city, Zhejiang province, to analyze their unmet needs in health and health services from the perspective of the demand side of health services (this study refers to older adults in the community) and suggest how the supply side of health services (this study relates to medical and health institutions at all levels) should better integrate their resources. This provides ideas and suggestions for the design of the organizational structure of an integrated health care delivery system.

## Method

### Study design and participants

Taking the elderly in District N of H City, Zhejiang Province, China, as the health service demand-side research object, the permanent elderly in three streets (townships) were randomly selected by casting lots through a group sampling method. Inclusion criteria: (1) Age ≥ 60 years old (2) Clear-mindedness and ability to answer questions correctly (3) Voluntary participation in each questionnaire and signing of informed consent. Exclusion criteria: Non-resident population of the district. A total of 1527 cases were enrolled.

This study was conducted through a questionnaire survey. *The Household Health Questionnaire for Investigation Chronic Diseases and Their Risk Factors was used uniformly.* The questionnaire consists of the family's actual situation, the individual's actual situation, the primary chronic diseases and medical treatment, behaviors and lifestyles, the knowledge of chronic disease prevention and control, and the medical examination form.

## Data collection

A trained investigator fills out an interview based on the questionnaire content and patients' responses to ensure the accuracy and reliability of the filled-in. The questionnaire survey is done the on-site investigation, on-site filling, and on-site recycling. A total of 1527 questionnaires were distributed, and 1527 were recovered, with a recovery rate of 100%. Among them, 1447 valid questionnaires were recovered, and the effective recovery rate was 94.8%.

## Statistical methods

The questionnaires collected were encoded, and all eligible data were established in a database using EpiData3.1 and entered by two people. SPSS25.0 is used for statistical analysis, and the counting data is expressed as frequency and percentage.

## Results

### Demographic data and general characteristics

Table 1 shows demographic data and general characteristics of the participants. Of 1447 elderly, men and women accounted for 46.4 percent and 53.6 percent, respectively. The primary school and below population accounts for 86.5 percent, and the health insurance coverage is 100 percent. Overweight accounted for 29.8%.

**Table 1:** Demographic data and general characteristics

project	frequency
Gender	N(%)
man	672(46.4)
woman	775(53.6)
Education	
illiteracy	507(35)
elementary school	745(51.5)
Junior high school and above	195(13.5)
Health insurance	
Publicly funded medical care	9(0.6)
Basic medical insurance for urban and rural residents	1438(99.4)
BMI	
< 18.5	89(6.1)
18.5–23.9	927(64.1)
≥ 24	431(29.8)
Total	1447(100)

## Prevalence of chronic diseases

Table 2 shows the prevalence of chronic diseases. The top three diseases with chronic disease prevalence were hypertension (40.2%), dyslipidemia (8.4%), and diabetes mellitus (7.0%). Many older adults do not know their diseases, of which 2.8%, 1.7%, 3.2%, or 5.7% of the elderly do not know whether they have coronary heart disease, stroke, chronic respiratory diseases, and cancer, respectively.

**Table 2:** Prevalence of chronic diseases

project	frequency
Hypertension	N(%)
yes	582(40.2)
I don't know	5(0.3)
Diabetes	
yes	102(7.0)
I don't know	16(1.1)
Dyslipidemia	
yes	122(8.4)
I don't know	245(16.9)
Coronary heart disease	
yes	61(4.2)
I don't know	40(2.8)
Stroke	
yes	17(1.2)
I don't know	25(1.7)
Chronic respiratory diseases	
yes	38(2.6)
I don't know	47(3.2)
Cancer	
yes	13(0.9)
I don't know	83(5.7)

## Unmet needs of health service demanders to obtain disease-related knowledge and lack of health concepts

Table 3 shows that health service providers' demand for disease-related knowledge is unmet and lacks a health concept. 74.5% of the elderly do not know the standards of the high-risk group of chronic diseases, 31% do not know which conditions are chronic, and only 18.7% of the elderly know their blood glucose levels, indicating that the awareness rate of chronic disease-related knowledge is low and the demand for disease-related knowledge Not satisfied. Only 27% of patients with dyslipidemia have been treated with lipid-modulating drugs in the past two weeks, with low medication compliance and unmet needs for safe medication.

**Table 3:** Health literacy – basic knowledge and philosophy

project	Frequency N(%)
I don't know the criteria for people at risk for chronic diseases	1078(74.5)
I don't know which conditions are chronic	448(31.0)
I don't know what factors are associated with the development of chronic diseases	426(29.4)
Believes that a healthy lifestyle can effectively prevent chronic diseases	965(66.7)
The most critical thing to prevent and control cardiovascular diseases is to prevent and control risk factors such as high blood pressure	860(59.4)
Most cancers are considered preventable and treatable	523(36.1)
The prevention and control of chronic diseases are considered to require the participation of the whole society	914(63.2)
The hypertensive person taking antihypertensive medication within the last two weeks	544(93.5)
Diabetic treated with glucose-lowering medication or insulin within the last two weeks	89(87.3)
Dyslipidemia treated with lipid-regulating drugs within the last two weeks	33(27.0)
Know your waistline	943(65.2)
Know your blood pressure	1069(73.9)
Know your blood sugar	270(18.7)

## Health services demand reasonable side exercise, diet, and nutrition needs are not met, lack of healthy lifestyles and behavior

As seen in Table 4, the demand for rational exercise, diet, and nutrition on the health service side is not being met, and there is a lack of healthy lifestyles and behaviors. Smoking and alcohol consumption accounted for 21.2% and 18.3%, respectively. Only 17.9% of people exercise in their spare time, lack of exercise and suitable sports needs are not met. The proportion of not eating fish, shrimp, eggs, and fruits every week is about 10%, and the balance of not eating dairy products every week is as high as 84.9%. The dietary structure is unreasonable, and the dietary nutritional needs are unmet. Only 57.3% of the elderly will take the initiative to go to medical institutions after becoming ill, and they lack awareness of active medical treatment.

**Table 4:** Health literacy – lifestyle and behavior

project	frequency
Whether or not to smoke	N(%)
smoking	307(21.2)
Have quit smoking	131(9.1)
Whether to drink alcohol	
Drinking	265(18.3)
Have quit drinking	77(5.3)
Exercise in your spare time	259(17.9)
walk	671(46.4)
Ride a bicycle	135(9.3)
Do housework	936(64.7)
Do not eat pork, beef, and poultry meat every week	55(3.8)
Do not eat fish and shrimp every week	106(7.3)
Do not consume eggs weekly	125(8.6)
No fruit consumption per week	170(11.7)
Do not consume soy products every week	267(18.5)
Do not consume dairy products weekly	1229(84.9)
Do not consume nuts weekly	1301(89.9)
After falling ill, he went to a medical institution for treatment	829(57.3)

## Health service demand-side health information access, use of unmet needs, lack of basic health skills

Table 5 illustrates the unmet needs for health information acquisition and utilization of health service needs and the lack of basic health skills. About 70% of the elderly do not know the daily intake of salt and edible oil, and only 10.3% of the elderly pay attention to the nutrition labels on the food packaging when they buy food. Unmet need for health information access and utilization.

**Table 5:** Health literacy – essential skills

project	Frequency N(%)
Know the daily salt intake of healthy adults	535 (37.0)
Know healthy adults daily cooking oil intake	390 (27.0)
Know food nutrition labels	176 (12.2)
When buying food, you will look at the food nutrition label on the bag	149 (10.3)

## Discussion

For a long time, the standardized health management of the elderly in the community has generally been based on three basic steps: understanding the health status, conducting health and disease risk assessments, and conducting health interventions [11]. However, there is no specific description of which level of health service will be completed by each step. Some scholars have proposed that the health management of the elderly should be carried out with primary medical and health institutions as the main body [12]. In recent years, the government has responded to the increasing aging of the population and reduced the burden of non-communicable diseases represented by chronic diseases through functional complementarity [13], knowledge sharing [14], and integration of primary health services with public health services [15] at all levels of health care services in the region. However, Chinese primary health care institutions still suffer from insufficient allocation of quality resources and inflexible operating mechanisms, which restrict the capacity of primary health care services [16], and the health service needs of the elderly in the community are not met.

In the health service system, general practitioners play the role of "gatekeepers". In contrast, in medical and health service institutions at all levels, secondary medical and health service institutions are "carrying on from top to bottom." To better meet the growing demand for health services for the elderly in the community, we recommend that general practitioners be organically combined with secondary medical and health service institutions, with known practitioners as the hub. They give full play to the role of general practitioners as experts and gatekeepers in secondary medical and health service institutions [17]. Next, the development of a cluster model centered on general practitioners continues [18, 19]. Services such as lifestyle and behavioral counseling, health assessment, chronic disease management, and diagnosis and treatment of common diseases are provided to the elderly. Following a person-centered and population-based approach [6], they are responsible for monitoring the health status of older adults in the community, conducting routine physical examination practices, and performing early screening for diseases to provide older adults with an initial understanding of their health status. Determine referral decisions [20], and maintain good communication and interaction with medical specialists when making referrals [21]; Conduct health promotion programs [22]; Bring in outside professionals to provide the personal level of support services needed by older adults [23]. At the top, the General Practice Liaison (GPL) service is actively developed to improve the exchange and transfer of information between GPs and hospitals [24]. Sharing of information such as screening and treatment [25]. Make full use of the resources of the specialist's workshop [26] set up by the higher-level medical institutions to provide more authoritative treatment plans and more detailed lifestyle and behavioral guidance for the elderly. Institutions to offer more authoritative treatment plans and more detailed lifestyle and behavior advice for the elderly. Assist in taking charge of the operational training of medical staff and quality control of medical services in lower-level medical institutions, so that medical institutions at all levels can achieve homogenization of services [27]. With the development of information technology, it is necessary to give full play to the advantages of network technology when carrying out various services [28]. At the same time, pay attention to avoiding medical data leakage and improving the security and confidentiality of medical data [29, 30]

For the demand for health services, other studies are more from the perspective of health service providers. Our research directly dialogues with the demand side, from the demand side, analyzes their current unmet needs, and proposes possible solutions, providing new ideas for such research. Our study has certain limitations. The sample source is relatively single, only collected the data of the elderly in the N district of H City, Zhejiang Province, China. With local particularity, the sample size can be expanded in the future, collect and analyze the unmet needs of health service needs in different regions, and propose more targeted solutions.

## Conclusion

From the perspective of meeting the balance between supply and demand of health services, the health service system should break the concept of the original three-tier medical system hierarchy. In China's current health care system, secondary health care institutions are in the "middle" and general practitioners have the role of "gatekeepers". We propose to take GPs in secondary medical and health service institutions as the hub and use the integrated medical and health service system as the working platform for GPs, and give them the authority to provide primary care, health consultation, community rehabilitation, referral decision, and service liaison, etc. At the same time, medical institutions at all levels should link up and do their own work, with the core of meeting the health service needs of the elderly in the community, and give full play to the role of GPs based on their own functions and positioning. In order to achieve the goal of "Healthy Aging", we will fully utilize the role of general practitioners and make full use of the resources of integrated medical care.

## Supplementary Information

**Additional file 1.**

**Additional file 2.**

**Additional file 3.**

## Data Availability

All data generated or analysed during this study are included in this published article [and its supplementary information files].
