# Supplementary material for: Can the organization of health resource integration be analyzed in terms of the current state of unmet demand for health services? take the health needs of the elderly in a place in zhejiang province, china, as an example
Source: BMC Prim Care. 2022 Nov 19;23:288. doi: 10.1186/s12875-022-01893-7 (PMC9675962; doi:10.1186/s12875-022-01893-7)
Supplement: Supplementary file 1 — Additional file 1. [file 12875_2022_1893_MOESM1_ESM.docx]

Description of relevant data in EXCLE table

Gender：1.man 2.woman

Education：1.illiteracy 2.elementary school 3and above.Junior high school and above

Health insurance:2.Publicly funded medical care other options.Basic medical insurance for urban and rural residents

Hypertension:1.Yes 2.No 9.I don't know

Diabetes:1.Yes 2.No 9.I don't know

Dyslipidemia:1.Yes 2.No 9.I don't know

Coronary heart disease:1.Yes 2.No 9.I don't know

Stroke:1.Yes 2.No 9.I don't know

Chronic respiratory diseases:1.Yes 2.No 9.I don't know

Cancer:1.Yes 2.No 9.I don't know

Do you know the criteria for people at high risk of chronic diseases:1.Yes 2.No

Do you know which of the following diseases are chronic:1-4Names of different diseases 9.I don't know

Do you know what factors are associated with chronic disease:1-8Names of different factors 9.I don't know

Do you think a healthy lifestyle can effectively prevent chronic diseases:1.Yes 2.No 9.I don't know

Do you think prevention and treatment of cardiovascular and cerebrovascular diseases is most important to prevent and control hypertension and other risk factors:1.Yes 2.No 9.I don't know

Do you think most cancers are curable:1.Yes 2.No 9.I don't know

Do you think that the prevention and control of chronic diseases need the participation of the whole society:1.Yes 2.No 9.I don't know

The hypertensive person taking antihypertensive medication within the last two weeks:1.Yes 2.No

Diabetic treated with glucose-lowering medication or insulin within the last two weeks:1.Yes 2.No

Dyslipidemia treated with lipid-regulating drugs within the last two weeks:1.Yes 2.No

Know your waistline:1.Yes 2.I don't know

Know your blood pressure:1.Yes 2.I don't know

Know your blood sugar:1.Yes 2.I don't know

Whether or not to smoke:1.smoking 2.Have quit smoking 3.Never smoking

Whether to drink alcohol:1.Drinking 2.Have quit drinking 3.Never drinking

Exercise in your spare time:1.Yes 2.No

Walk:1.Yes 2.No

Ride a bicycle:1.Yes 2.No

Do housework:1.Yes 2.No

Whether to treat after illness:1.Yes,Self-treatment 2.Yes,Go to a medical institution for treatment 3.No

Daily salt intake for healthy adults:Choice 2 is correct

Daily cooking oil intake for healthy adults:Choice 2 is correct

Know food nutrition labels:1.Yes 2.No

When buying food, you will look at the food nutrition label on the bag:1.Yes 2.No
